# The effect of 8-week combined balance and plyometric on the dynamic balance and agility of female adolescent taekwondo athletes

**DOI:** 10.1097/MD.0000000000037359

**Published:** 2024-03-08

**Authors:** Xiang Shen

**Affiliations:** a School of Physical Education, Guangzhou College of Commerce, Guangzhou, Guangdong, China.

**Keywords:** agility, combined training, dynamic balance, female, taekwondo

## Abstract

**Objectives:**

To investigate the effect of combined balance and plyometric training (PT) on the agility and dynamic balance of adolescent taekwondo athletes.

**Methods:**

Thirty female adolescent taekwondo players volunteered to participate and were randomly assigned to the combined balance training and PT (CT; n = 15) and PT (n = 15) groups. The CT group performed balance training combined with PT 3 times a week for 6 weeks (40 minutes of plyometrics and 20 minutes of balance training) while the PT group performed only PT for the same period (3 sets × 8–12 reps for each exercise). Both groups received the same routine technical taekwondo training.

**Results:**

Post-intervention assessments revealed that both groups significantly improved their dynamic posture stability index scores (DPSI; forward jump [F-DPSI] and lateral jump [L-DPSI]). However, participants in the CT group achieved notably superior outcomes in the F-DPSI and L-DPSI scores compared with those achieved by their PT counterparts. The center of pressure metrics exhibited improvements post-intervention, with scores of specific measures in the PT group surpassing those in the CT group. Additionally, the 5-0-5 test scores exhibited improvements post-intervention, with scores of specific measures in the PT group surpassing those in the CT group, and the TAST (Taekwondo Specific Agility Test) of the CT group and the PT changed significantly after the intervention.

**Conclusion:**

An 8-week regimen that integrates balance and plyometric training effectively augments knee function and proprioception in adolescent Taekwondo athletes. This study underscores the potential benefits of a combined training approach, providing coaches and athletes with valuable insights into Taekwondo training.

## 1. Introduction

Agility and lower dynamic balance are critical factors influencing the performance and career longevity of adolescent Taekwondo athletes.^[1]^ Enhanced performance parameters and minimized injury risk can substantially contribute to athletes’ overall efficacy in sports.

Plyometric training (PT), a popular strategy designed to improve lower limb strength, knee function, and landing movement patterns, has been widely investigated in this context.^[2]^ PT enhances performance by shortening the eccentric-concentric muscle contraction cycle, also known as the stretch-shortening cycle.^[3]^ Empirical evidence has shown that PT can optimize sports performance attributes, such as agility,^[4]^ and even reduce the risk of injuries such as injury to the anterior cruciate ligament (ACL).^[5]^

The efficacy of PT has recently been enhanced through its combination with other training programs. Research has indicated that such combined training (CT) programs could substantially enhance the benefits of PT, particularly in dynamic balance.^[6,7]^ Studies have demonstrated that CT can target various mechanisms that contribute to knee function and proprioception, as well as enhance reflexive responses and assist body positioning adjustments during landings. These benefits are particularly important for reducing the incidence of injuries, as evidenced by a study involving young female handball players.^[8]^

Despite the promising findings on CT, comprehensive research examining the impact of CT on agility and lower limb injury risk in elite adolescent Taekwondo practitioners is lacking. Furthermore, it remains unclear whether CT confers greater benefits than PT alone. Therefore, our pilot randomized controlled study sought to elucidate the effects of an 8-week CT program, which integrates balance training and PT, on knee function and proprioception in a cohort of elite adolescent Taekwondo athletes. We hypothesized that the use of CT would improve the agility and dynamic balance of taekwondo athletes better than PT alone.

## 2. Materials and methods

The participant sample size (n = 24) was calculated employing G*Power software (version 3.1.9.7; Franz Faul, University of Kiel, Germany). This determination utilized the following parameters: alpha error probability (α) set at 0.05, power (1-β error probability) at 0.8, effect size (f) at 0.4, test family as F-tests, and the statistical test specified as analysis of variance (ANOVA) for repeated measures, encompassing within-between interaction.^[9]^ This study recruited 30 elite adolescent female taekwondo athletes. The inclusion criteria were as follows: Participants who had secured positions among the top 4 in the national youth games, provincial games, or games of higher prestige. Participants whose dominant arm or leg is on the right side. Participants who were capable and willing to commit to the 8-week program encompassing tests and interventions.

Conversely, exclusion criteria encompassed: A history of significant lower extremity trauma, including injuries to the anterior cruciate ligament, hamstring, meniscus, or ankle within 3 years preceding the study. A Limb Symmetry Index below the 85th percentile threshold as determined by single-legged hop assessments.

Within the cohort, 8 athletes had progressed to the quarterfinals of national youth competitions, and 22 had attained finalist positions at the provincial level. All participants were affiliated with the same training institution. Their regimen included thrice-weekly sessions, each extending from 2 to 3 hours, dedicated to both technical skill enhancement and physical conditioning.

The study protocol received endorsement from the Research Ethics Board of Guangzhou College of Commerce (Approval number: 2023001), aligning with the ethical guidelines enumerated in the Declaration of Helsinki. Preliminary to data acquisition, a detailed orientation regarding the prospective benefits and inherent risks of the study was provided to all participants. Ensuingly, written informed consent was procured from each participant, affirming their informed and voluntary engagement in the research activities.

### 2.1. Procedures

**Figure 1.**
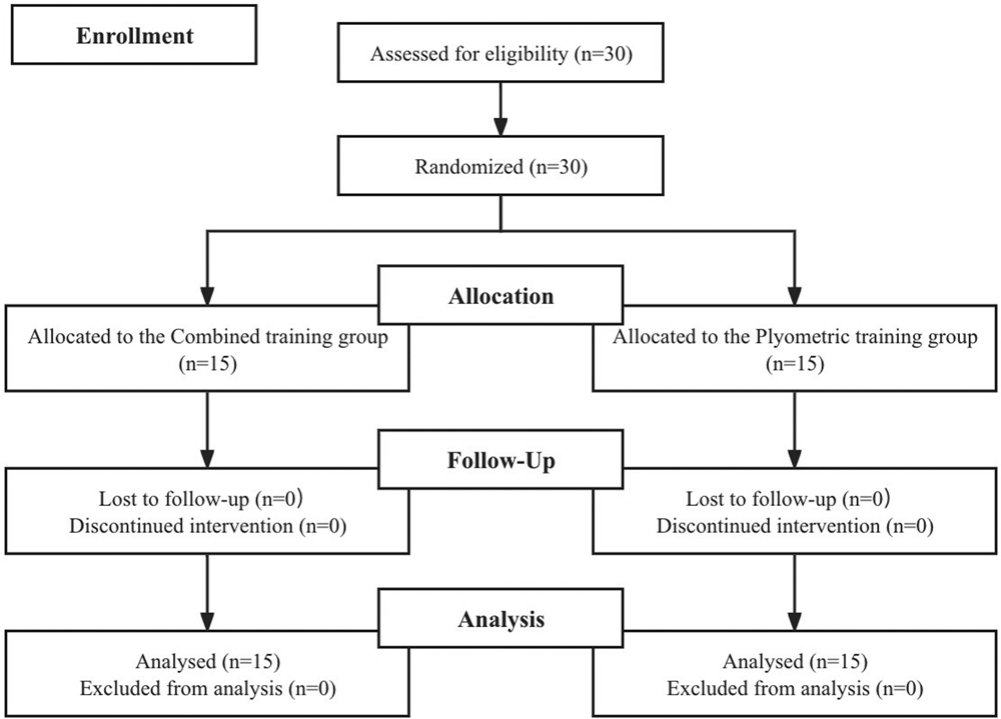
Flow chart of the progress.

The experimental training regimens were systematically integrated with routine technical training on a weekly basis. Study subjects were recruited from July August10 to August 20, 2023, and the experiment was conducted from August 21 to October 21. Participants were allocated randomly to either the Combined Training (CT, n = 15) or Plyometric training (PT, n = 15) groups according to a computer-generated randomization list, as delineated in Table 1 and Figure 1. Prior to the commencement of the primary study, a 2-week acclimatization phase was undertaken, comprising 3 sessions weekly, to familiarize participants with the training protocols subsequently employed in the intervention. During the intervention phase, the CT cohort engaged in a composite of balance and PT exercises. This entailed 3 weekly sessions of CT over an 8-week span, totaling 18 sessions. Each CT session encompassed 40 minutes of PT—incorporating exercises such as depth jumps and lateral barrier jumps—followed by 20 minutes of balance training utilizing unstable platforms including BOSU balls, Swiss balls, and balance pads. Conversely, the PT cohort adhered to a regimen of 3 PT sessions per week, also spanning 8 weeks. To equilibrate the training volume across both cohorts, PT participants performed 40 minutes of PT, subsequently engaging in 20 minutes of balance training on stable surfaces, such as a solid floor. Inter-session intervals were maintained at 24 to 48 hours. The comprehensive protocol for balance training and PT is explicated in the Supplementary Material, Tables S1 and S2; http://links.lww.com/MD/L810; http://links.lww.com/MD/L811. The refinement of this training protocol was informed by a review of pertinent literature.^[7]^

**Table 1:** The descriptive characteristics of the participants.

	Age (yr)	Height (cm)	Weight (kg)	Training experience (yr)
PT(n = 15)	15.93 ± 0.88	177.20 ± 4. 49	68.53 ± 4.00	5.60 ± 0.83
CT(n = 15)	15.80 ± 0.68	177.80 ± 3.30	66.93 ± 3.99	5.80 ± 0.86

### 2.2. Dynamic posture stability test

The examination was designed to evaluate the athletes’ dynamic balance by quantifying the Dynamic Posture Stability Index (DPSI) and the Center of Pressure (COP) trajectory.^[10]^ Participants performed this assessment by initiating from a standing position on an embedded force platform (Kistler 9281CA, KISTLER, Winterthur, Switzerland, sampled at 1000 Hz). The protocol involved a forward or lateral jump using the dominant leg, followed by a 10-second single-leg stance on the same leg upon landing. The jump origination point was calibrated to 40% of the athlete stature in centimeters, a distance that was meticulously maintained from the center of the force plate.^[10,11]^ A barrier was strategically placed at the midpoint along the axis connecting these 2 points, with the barrier elevation adjusted to 30 cm for forward jumps and 15 cm for lateral movements. Participants were required to perform both jump variations in triplicate, with the mean of these trials subjected to further analytical processing. The analysis employed Matlab software (r2014b, MathWorks, Natick, MA) to compute the DPSI and COP values. Time-series recordings of the ground reaction forces (GRF) and COP were captured during the 10-second interval post-landing. Subsequently, data refinement was performed using low-pass filter processing with a cutoff frequency established at 13.33 Hz.


$$\begin{array}{l} DPSI=\frac{\left(\frac{\sqrt{\sum {\left(0-GRF_{x}\right)}^{2}+\sum {\left(0-GRF_{y}\right)}^{2}+\sum {\left(BW-GRF_{z}\right)}^{2}}}{\;\;\;number\;\;\;of\;\;\;data\;\;\;points\;\;\;}\right)}{BW} \end{array}$$

DPSI was calculated from the GRF curve within 3 seconds after touchdown (the time when the GRF value exceeded 5% of the body weight),^[12]^ where BW is the body weight and GRFx, GRFy, and GRFz are the back and forth, left and right, and vertical ground reaction forces, respectively. The DPSI of forward jump (F-DPSI) and lateral jump (L-DPSI) were calculated.

COP was calculated from the time series within 10 seconds after landing, and the back and forth displacement difference (COPAP), left-right displacement difference (COPML), and total displacement distance (COPPL) of forward jump (F) and lateral jump (L) were calculated.^[13]^


$$\begin{array}{l} COP_{AP}=\underset{1}{\overset{10}{\sum }}\;{\left(x_{t}-\bar{x}\right)}^{2} \end{array}$$

$$\begin{array}{l} COP_{ML}=\underset{1}{\overset{10}{\sum }}\;{\left(y_{t}-\bar{y}\right)}^{2} \end{array}$$

$$\begin{array}{l} COP_{PL}=\underset{0}{\overset{9}{\sum }}\;\sqrt{{\left(x_{t+1}-x_{t}\right)}^{2}+{\left(y_{t+1}-y_{t}\right)}^{2}} \end{array}$$

where xt and yt are the back and forth and left and right displacements, respectively at t seconds, and the value of t is 1 to 10 seconds.

### 2.3. 5-0-5 change of direction test

The 5-0-5 change of direction test is a standardized assessment tool utilized in disciplines such as basketball and football to quantify an athlete capacity for rapid directional changes and acceleration across brief spans.^[14]^ This test requires the symmetrical arrangement of 2 cones, designated as “A,” at the commencement line. An additional pair of cones, marked as “B,” is positioned 3 meters to the right and in alignment with point “A.” Similarly, a third cone, indicated as “C,” is situated 3 meters to the left and in parallel with point “A.” The timing of the test is automated by the Smart Speed system (Fusion Sport, Coopers Plains, Australia), strategically placed behind each cone pair. Participants were directed to initiate a rapid turn to the right and sprint from point “A” to “B” upon the auditory signal command “Ready, go!” Upon reaching point “B,” they were required to execute an immediate turn and sprint towards point “C,” followed by a final pivot and return sprint to point “A.” Each participant completed 3 attempts of the test, with the maximal score from the trials being recorded as the valid result. A rest interval of 5 to 10 minutes was mandated between successive trials.

### 2.4. Taekwondo specific agility test (TSAT)

The assessment of specific agility was conducted via the TSAT, in line with extant methodological guidelines.^[15]^ Participants commenced from a defensive stance with both feet retracted behind the demarcated start/finish line. The sequence of maneuvers required the athlete to: advance expeditiously to the central marker while maintaining the guard position and avoiding foot crossover; execute a pivot to confront the first sparring partner and deliver a roundhouse kick with the left extremity, termed a leading-roundhouse kick (dollyo tchagui); progress towards the second partner to execute a corresponding right-legged roundhouse kick (dollyo-chagi); revert to the central position; advance again in guard position to perform a double-roundhouse kick (narae-chagi) targeting the third partner; and retreat to the start/finish line in guard position. The first and second sparring partners were equipped with a single kick-target, while the third held dual targets, all of which were to be aligned with the athlete torso level. noncompliance with the protocol, such as feet crossing during movement or inadequate target contact, necessitated trial invalidation and a subsequent 3-minute rest before reattempt. The performance metric was the completion time, measured using a precision electronic timing system (Brower Timing Systems, Salt Lake City, UT). Athletes undertook 2 attempts, with the superior performance being retained for analysis, demonstrating intraclass correlation coefficients (ICCs) of 0.90 (95% confidence interval, CI: 0.87–0.92) pretest and 0.86 (95% CI: 0.78–0.87) post-test.

### 2.5. Statistical analysis

The experimental datasets were analyzed utilizing the IBM SPSS Statistics software suite (version 25.0, IBM, Chicago, IL). Descriptive statistics were represented by means and standard deviations (SD). A threshold of *P* < .05 was predetermined for statistical significance across all analytical tests. To investigate the impact of Combined Training (CT) on the outcomes of single-legged hop and proprioception evaluations, a preliminary 2-way repeated-measures analysis of variance (ANOVA; group × time) was employed. The response variables incorporated into each model were Frontal Dynamic Posture Stability Index (F-DPSI), Lateral Dynamic Posture Stability Index (L-DPSI), Frontal Center of Pressure in Anteroposterior (F-COPAP) and Mediolateral (F-COPML) and Path Length (F-COPPL), Lateral Center of Pressure in Anteroposterior, Mediolateral (L-COPML), and Path Length (L-COPPL), as well as the 5-0-5 change of direction test, and the Taekwondo-Specific Agility Test (TSAT). The factors introduced into the model were group, time, and their interaction. Upon detecting a significant interaction effect, a Least Significant Difference (LSD) post hoc adjustment was applied to elucidate the specific origins of the observed statistical significance. Subsequently, to discern the effects of CT within each cohort and to compare the proportional changes from baseline to post-intervention between the CT and PT groups, separate one-way ANOVA tests were conducted, with “time” as the independent variable, the factors introduced into the model was time. The magnitude of the observed effects was quantified using partial eta squared (Partial η^2), with effect size (ES) interpretations classified as follows: values below 0.06 indicating a small effect, values below 0.14 suggesting a moderate effect, and values of 0.14 or above denoting a large effect.^[16]^

## 3. Results

### 3.1. Dynamic balance ability

#### 3.1.1. Dynamic posture stability index.

The results of the F-DPSI and L-DPSI ANOVA models showed the significant effects of time [F-DPSI: F(1,56) = 41.466, *P* < .001, partial η^2^ = 0.425; L-DPSI: F(1,56) = 102.58, *P* < .001, partial η^2^ = 0.647]. This implies that compared with baseline, participants in both groups had significantly lower F-DPSI and L-DPSI scores after the intervention. A significant interaction between group and time [F-DPSI: F(1,56) = 4.319, *P* = .42, partial η^2^ = 0.72;L-DPSI: F(1,56) = 5.073, *P* < .001, partial η^2^ = 0.083] was also observed (Table 2). The post hoc analysis revealed that the participants who received CT had significantly lower F-DPSI (*P* < .001) and L-DPSI (*P* < .001) scores after the intervention compared with their baseline performance and those had by the participants who received PT. No significant group effects were observed (F-DPSI: *P* = .641; L-DPSI: *P* = .059).

**Table 2:** The assessment results for PB group and PT group before and after the 8-wk training.

		PB (N = 15)			PT (N = 15)		
		Pre	Post	Partial η^2	Pre	Post	Partial η^2
Dynamic balance	F-DPSI	0.377 ± 0.003	0.370 ± 0.002*	0.413	0.375 ± 0.003	0.371 ± 0.001*	0.145
	L-DPSI	0.377 ± 0.002	0.370 ± 0.002*#	0.578	0.375 ± 0.003	0.371 ± 0.001*	0.356
	F-COPAP (cm)	87.15 ± 12.62	79.31 ± 8.78*	0.064	92.50 ± 12.54	86.79 ± 11.35	0.035
	F-COPML (cm)	77.67 ± 12.62	82.86 ± 7.16	0.030	83.71 ± 14.85	77.00 ± 9.58	0.055
	F-COPPL (cm)	121.57 ± 7.19	112.97 ± 8.37*#	0.079	126.81 ± 12.62	120.89 ± 13.55	0.039
	L-COPAP (cm)	79.65 ± 7.99	74.84 ± 10.36	0.033	81.32 ± 12.99	74.65 ± 13.52	0.002
	L-COPML (cm)	96.06 ± 11.28	90.20 ± 11.34	0.039	99.30 ± 8.18	83.84 ± 11.62*	0.218
	L-COPPL (cm)	132.38 ± 8.91	119.38 ± 9.21*	0.212	134.47 ± 7.70	122.95 ± 10.00*	0.180
Agility	5-0-5	2.73 ± 0.23	2.39 ± 0.16*#	0.287	2.63 ± 0.23	2.56 ± 0.26	0.017
	TSAT	8.03 ± 0.44	7.39 ± 0.33*	0.249	8.27 ± 0.41	7.61 ± 0.42*	0.264

between PB group and PT group, *P* < .05. F, forward jump; L, lateral jump; COP, center of pressure, F-COPAP = frontal center of pressure in anteroposterior, F-COPML = frontal center of pressure mediolateral length, F-COPPL = frontal center of pressure path length, F-DPSI = frontal dynamic posture stability index, L-COPAP = lateral center of pressure in anteroposterior, L-COPML = lateral center of pressure mediolateral length, L-COPPL = lateral center of pressure path length, L-DPSI = lateral dynamic posture stability index, TSAT = taekwondo specific agility test.

* Statistically significant difference between pre- and post-test, *P* < .05.

# Statistically significant difference.

#### 3.1.2. Center of pressure.

Significant effects of time and group and non-significant interaction between group and time on F-COPAP [time: F(1,56) = 5.686, *P* = .021; group: F(1,56) = 5.098, *P* = .028] and F-COPPL [time: F(1,56) = 6.795, *P* = .012; group: F(1,56) = 5.590, *P* = .022] were observed. Specifically, compared with baseline, the F-COPAP, F-COPPL, and L-COPPL scores were significantly lower after the intervention, and these outcomes in the PT group were significantly greater than those in the CT group. Additionally, significant effects of time (F-COPML: *P* = .032; L-COPML: *P* < .001; L-COPPL: *P* < .001) but not group (F-COPML: *P* = .211; L-COPML: *P* = .573; L-COPPL: *P* = .210) and the interaction between group and time (F-COPML: *P* = .734; L-COPML: *P* = .088; L-COPPL: *P* = .789) were observed. No significant effects of time, group, and the interaction between group and time on lateral center of pressure in anteroposterior (*P* > .05) were observed.

#### 3.1.3. Agility performance.

A significant time [F(1,56) = 0. 614, *P* < .001, partial η^2^ = 0.226] and interaction between group and time [F(1,56) = 7.136, *P* = .010, Partial η^2^ = 0.113] but not group (*P* = .512) effects on 5-0-5 test scores were observed.

Significant effects of time and group [time: F(1,56) = 38.631, *P* < .001; group: F(1,56) = 4.879, *P* = .031] but not the interaction between group and time on TAST were observed.

## 4. Discussion

The objective of the present study was to scrutinize the impact of an 8-week regimen integrating balance training with proprioceptive training (PT) on agility and dynamic balance among female adolescent Taekwondo competitors. According to the extant literature, this research is pioneering in contrasting the outcomes of combined training modalities with PT exclusively within this athletic demographic. The results indicate that the inclusion of balance training in conjunction with PT confers enhanced improvements in knee joint function and agility when contrasted with PT in isolation.

The results of the investigation suggest that an 8-week period of CT led to more pronounced improvements in agility. Given that optimal agility necessitates robust lower limb muscular power for swift movement, as well as proficient balance for postural regulation and inertia mitigation during decelerations and abrupt halts, the integration of balance training into proprioceptive training was pivotal in enhancing these performance attributes.^[17,18]^ In the realm of combat sports, agility stands out as a critical and multifaceted skill, particularly integral to high-performance Taekwondo. This attribute enables athletes to execute technical and tactical maneuvers across various directions, thereby establishing it as a fundamental prerequisite for attaining peak performance levels.^[15,19]^ Agility is defined as the ability to rapidly change one body position through a series of movements, all the while maintaining appropriate control and balance.^[19]^ Meanwhile, although sufficient investigations have already evidenced positive adaptations in the COD ability of athletes after PT,^[20,21]^ the current study provided novel findings that CT could induce greater improvements. This is consistent with the findings of Guo et al^[18]^ and Bouteraa et al who applied a similar training methodology to badminton and female basketball players, respectively.^[22]^

The ability of players to maintain landing stability and balance is reflected in the DPSI and COP scores.^[12,23]^ The traditional PT often focuses on only musculoskeletal function, while the CT can simultaneously target multiple aspects pertaining to dynamic balance, such as the integration of visual and vestibular information and the coordination across these systems.^[24]^ Thus, this can benefit the capacity of the postural control system to appropriately reweight and utilize different types of sensory inputs (e.g., visual and proprioceptive) when receiving challenges or perturbations. For example, Nepocatych et al^[25]^ observed that balance training had the potential to induce adaptive responses in the neuromuscular system that enhances postural control, balance in women, and improvement of COP to effectively prevent chronic ankle instability. Additionally, previous studies have demonstrated that balance training leads to spinal reflex during postural movement, which results in less destabilizing movement.^[26]^ This increased ability can help improve ankle stability in adolescents, as Chronic ankle instability (CAI) was found to be prevalent in 20% of adolescent athletes.^[27]^ Therefore, the CT strategy would be more appropriate for achieving dynamic balance by simultaneously enhancing multiple underlying elements of dynamic balance control. This is extremely important for taekwondo athletes.

This study provides valuable insights into the effects of Combined Training (CT) on agility and dynamic balance in female Taekwondo athletes, despite some limitations due to the small and specific sample size. The focus on elite female adolescents, influenced by their intensive training and competition schedules, underscores the need for future research to expand the participant base. Including a broader and more varied group of athletes, both male and female across different age categories, will enhance and confirm these initial findings. Exploring the optimal intensity and the dose-response relationship of CT remains a promising direction for future studies. Incorporating more regular assessments and extended follow-up will help understand the long-term benefits of CT and optimize its application. Additionally, future studies should consider adding a nonintervention control group alongside the PT training group to gain deeper insights into the benefits of CT. Advanced analytical techniques focusing on the musculoskeletal and sensory systems will be crucial in these studies. They will not only validate the effectiveness of CT compared to single-modality training but also shed light on how CT improves dynamic balance and agility. This knowledge will contribute to developing more effective training protocols in Taekwondo and related sports. The findings highlight the significance of incorporating balance training and proprioception exercises into athletic training for enhanced knee function and proprioception, underscoring the value of integrative training approaches in sports.

## 5. Conclusion

An 8-week program combining balance training with proprioceptive training significantly enhances knee functionality and proprioceptive acuity in adolescent Taekwondo practitioners. The findings of this study highlight the efficacy of a Combined Training (CT) strategy, offering crucial insights for trainers and athletes to optimize Taekwondo training methodologies.

## Author contributions

**Conceptualization:** Xiang Shen.

**Data curation:** Xiang Shen.

**Formal analysis:** Xiang Shen.

**Funding acquisition:** Xiang Shen.

**Investigation:** Xiang Shen.

**Methodology:** Xiang Shen.

**Project administration:** Xiang Shen.

**Resources:** Xiang Shen.

**Software:** Xiang Shen.

**Supervision:** Xiang Shen.

**Validation:** Xiang Shen.

**Visualization:** Xiang Shen.

**Writing – original draft:** Xiang Shen.

**Writing – review & editing:** Xiang Shen.

## Supplementary Material




