# Supplementary material for: The effect of 8-week combined balance and plyometric on the dynamic balance and agility of female adolescent taekwondo athletes
Source: Medicine (Baltimore). 2024 Mar 8;103(10):e37359. doi: 10.1097/MD.0000000000037359 (PMC10919456; doi:10.1097/MD.0000000000037359)
Supplement: Supplementary file 1 [file medi-103-e37359-s001.docx]

Supplementary Material

**Table S1.** The balance training program for balance-plyometric (combined training) (CT) group and plyometric training (PT) group.

| Exercises | The first stage (1 week) | The second stage (2-4 weeks) | The third stage (5-8 weeks) |
| --- | --- | --- | --- |
| Stand on the balance board exercise | Static standing on the board with two legs  (3 sets: 30 s/set) | Static standing on the board with two legs and eyes closed  (3 sets: 30 s/set) | Squat on the plate with eyes closed  (3 sets: 10 reps/set) |
| Supine straight leg bridge on Swiss Ball | Isometric supine straight leg bridge on Swiss Ball  (3 sets: 30 s/set) | Isometric supine single-leg bending bridge on Swiss Ball  (3 sets: 30 s/set) | Dynamic supine single-leg bending bridge on Swiss  (3 sets: 10 reps/set) |
| Side-plank with inflated balance disc | Side-plank with inflated balance disc with elbow  (3 sets: 30 s/set) | Side-plank with inflated balance disc and the non-supporting leg stretches backward  (3 sets: 10 reps/set) | Side-plank with inflated balance disc and the non-supporting leg stretches backward with elastic band  (3 sets: 10 reps/set) |
| Lunge squat on BOSU ball | Lunge squat on BOSU ball  (3 sets: 10 reps/leg/set) | Lunge squat on BOSU ball and inflated balance disc  (3 sets: 10 reps/leg/set) | Lunge squat on BOSU ball and inflated balance disc with 5 kg dumbbells  (3 sets: 10 reps/leg/set) |
| Airex® Balance-pad Elite exercise | Single-leg squat with balance-pad  (3 sets: 10 reps/leg/set) | Single-leg standing with balance-pad and the non-supporting leg stretches  backward  (3 sets: 12 reps/leg/sets) | Single-leg support with  balance-pad elite and the non-supporting leg stretches  backward with elastic band  (3 sets: 12 reps/leg/sets) |
| Rest | Between exercise: 60 s Between sets: 3 min | | |

Note: CT group conducted training program on unstable support (e.g., BOSU ball, Swiss ball, and Balance pad); and PT group conducted training program on stable support (i.e., solid floor).
