# Supplementary material for: The effect of 8-week combined balance and plyometric on the dynamic balance and agility of female adolescent taekwondo athletes
Source: Medicine (Baltimore). 2024 Mar 8;103(10):e37359. doi: 10.1097/MD.0000000000037359 (PMC10919456; doi:10.1097/MD.0000000000037359)
Supplement: Supplementary file 2 [file medi-103-e37359-s002.docx]

Supplementary Material

**Table S2.** The plyometric training (PT) program for CT and PT group.

| Exercises | The first stage (1 week) | The second stage (2-4 weeks) | The third stage (5-8 weeks) |
| --- | --- | --- | --- |
| Front barrier jump (6 hurdles) | Double-leg front barrier jump (15 cm)  (3 sets: 10 reps/set) | Single-leg front barrier jump (15 cm)  (3 sets: 5 reps/leg/set) | Single-leg front barrier jump (30 cm)  (4 sets: 5 reps/leg/set) |
| Lateral high-knees with hurdles | 4-hurdle (15 cm)  (3 sets: 2 reps/set) | 6-hurdle (30 cm)  (3 sets: 4 reps/set) | 6-hurdle (30 cm)  (3 sets: 6 reps/set) |
| Lateral barrier jump | Double-leg jump (15 cm)  (3 sets: 10 reps/set) | Double-leg jump (30 cm)  (3 sets: 12 reps/set) | Single-leg jump (30 cm)  (3 sets: 15 reps/leg/set) |
| Depth jump | Jump with 20 cm box  (3 sets: 8 reps/set) | Jump with 30 cm box  (3 sets: 8 reps/set) | Jump with 40 cm box  (3 sets: 8 reps/set) |
| Multi-direction jumps with hurdles | Triangle jump with double-leg (3 hurdles)  (3 sets: 6*3 reps/set) | Square jump with single-leg (4 hurdles)  (3 sets: 8*3 reps/set) | Hexagon jump with single-leg (6 hurdles)  (3 sets: 12*3 reps/set) |
| Intensity and number of contacts with ground | Low intensity  144 | Middle intensity  234 | High intensity  325 |
| Rest | Between exercise: 60 s Between sets: 3 min | | |
